# Seasonal variation in cutaneous melanoma incidence: link with recent UV exposure. A Belgian population-based study

**DOI:** 10.1186/2049-3258-73-S1-O1

**Published:** 2015-09-17

**Authors:** Kristine Rommens, David Jegou, Hugo De Backer, Joost Weyler

**Affiliations:** 1 Universiteit Antwerpen, Wilrijk, Belgium; 2 Belgian Cancer Registry, Brussels, Belgium; 3 Royal Meteorological Institute, Uccle, Belgium

## 

Our objective was to test the hypothesis of a short-term late-promoting effect of UV exposure on the development of cutaneous melanoma as explanation for the summer peak in melanoma incidence. Therefore, we studied seasonal variation in melanoma incidence in relation to recent UV exposure using direct UV measurements. Data from the Belgian Cancer Registry on invasive cutaneous melanoma diagnosed during 2006-2011 were used for analysis. Daily data on UV measurements in Belgium were obtained from the Royal Meteorological Institute. Simple and multiple negative binomial regression models were used to investigate the influence of recent UV exposure on melanoma incidence. The sum of the mean UV dose in the two months prior to diagnosis was used as a proxy for recent UV exposure in the population. To include variable sunburn risk during the year, the categorical variable ‘semester’ was created. The incidence of melanoma in Belgium shows a distinct seasonal variation with peaks in June or July. We found that part of this variation could be explained by the variation in dermatologic activity and therefore used this as an offset in our models. We found a linear relationship between melanoma incidence and UV dose in the two months preceding the diagnosis. UV exposure had more impact in the first semester. The effect of UV exposure was not modified by gender or age. The interaction between anatomical site and UV exposure was significant (p<0.001) and showed a higher effect on the upper and lower limbs compared to head and neck and trunk.

